# Unlocking the road to entrepreneurial success: quality drivers and digital competence in cloud computing adoption

**DOI:** 10.1038/s41598-026-41143-9

**Published:** 2026-03-06

**Authors:** Chun Wu, Ahmed Muneeb Mehta, Zhi Li, Muhammad Asif, Muhammad Farrukh Shahzad

**Affiliations:** 1 School of Continuing Education, NingboTech University, Ningbo, 315000 China; 2 Hailey College of Banking and Finance, University of the Punjab, Lahore, Pakistan; 3 Wittenborg University of Applied Sciences, Apeldoorn, Netherlands, Pakistan; 4 School of Media and Law, NingboTech University, Ningbo, 315000 China; 5 UE Business School, University of Education, Lahore, Pakistan; 6 College of Economics and Management, Beijing University of Technology, Beijing, 100124 PR China

**Keywords:** Cloud computing, SMEs, Information quality, Service quality, System quality, Artificial neural network, Energy science and technology, Engineering

## Abstract

Cloud computing has transformed small and medium-sized enterprises (SMEs) by providing flexible, scalable, and affordable IT solutions. However, adoption decisions are influenced by issues with digital competency, trust, and service quality; therefore, it’s critical to investigate the main factors that support and hinder cloud adoption in SMEs. Based on data from 367 managers in Pakistan, this study employs a hybrid structural equation modelling (SEM) and artificial neural network (ANN) approach to examine the effects of information quality, service quality, and system quality on cloud computing adoption in SMEs. Regulatory support as a mediator, and entrepreneurial digital competency as a moderator. The findings reveal that information quality and service quality significantly impact cloud computing adoption in SMEs, while system quality has an insignificant effect, and that regulatory support mediates these connections to some extent. Furthermore, entrepreneurial digital competency has a negligible effect on system quality but moderates the effects of information quality and service quality on cloud computing adoption. Regulatory support is the most significant predictor, according to ANN data. This study enhances the literature by using a hybrid SEM-ANN approach to investigate nonlinear interactions in SMEs’ adoption of cloud computing, offering fresh perspectives on the moderating role of entrepreneurial digital competency.

## Introduction

Cloud computing transforms SME operations through enhanced cost-effectiveness, scalability, and agility^1^. SMEs can greatly benefit from this technology, especially in Pakistan, where SMEs represent a significant portion of the economy. Adopting cloud computing can drive innovation and improve operational efficiency among these businesses^2^. However, SMEs often encounter challenges when implementing cloud solutions. Concerns related to data security, system reliability, service quality, and regulatory uncertainties can hinder their progress^3^. Examining these challenges more closely is essential to better understanding how quality considerations affect cloud adoption and how digital competency influences entrepreneurial decision-making.

Despite the numerous benefits of cloud computing, SMEs in Pakistan face several obstacles to adoption. Key challenges include issues related to information quality (IMQ), service quality (SRQ), and system quality (STQ). System inefficiencies, unpredictable service performance, and poor data accuracy hinder SMEs from fully benefiting from cloud-based solutions^4^. Additionally, the lack of legal clarity can increase the perceived risks associated with cloud services, making regulatory support (RLS) a crucial factor in cloud adoption decisions. Furthermore, managers’ ability to evaluate, adopt, and integrate cloud technology into their business processes is influenced by their level of entrepreneurial digital competency (EDC)^5^. However, how EDC moderates the relationship between quality criteria and cloud adoption, particularly in the SME sector of emerging nations such as Pakistan, remains underexplored in the literature.

SMEs in developing nations have received inadequate attention in the cloud computing literature, which has primarily focused on large corporations. Previous research examining quality characteristics such as IMQ, SRQ, and STQ as factors influencing technology adoption has largely overlooked the moderating role of EDC. Additionally, the mediating role of RLS, which is crucial for ensuring a secure and organised cloud computing environment, remains poorly understood. Moreover, the integration of advanced predictive analytics, such as ANNs, to model adoption decisions in SMEs is largely absent in existing studies, representing a methodological gap.

This study addresses these gaps by utilising the Technology-Organisation-Environment (TOE) framework. This framework provides a comprehensive understanding of how quality characteristics impact cloud adoption in SMEs, with RLS serving as a mediator and EDC serving as a moderator^6,7^. By simultaneously examining the combined effects of quality factors, regulatory support, and digital competency using both explanatory and predictive methods, this study extends existing knowledge. It offers a more holistic and actionable understanding of SME cloud adoption. By providing insights into the strategic facilitators and barriers to cloud adoption within Pakistan’s entrepreneurial landscape, this study contributes to both theoretical knowledge and practical applications.

This study aims to achieve two main objectives. First, it employs structural equation modelling (SEM) to examine how the adoption of cloud computing in SMEs is influenced by IMQ, SRQ, and STQ, with RLS as a mediator and EDC acting as a moderator. Second, it utilizes artificial neural networks (ANN) to develop a high-performance predictive model for SME managers’ decisions regarding cloud computing adoption (CCA). By integrating both explanatory and predictive approaches, this study provides a comprehensive framework for understanding and enhancing cloud adoption within Pakistan’s entrepreneurial ecosystem. In line with the study’s objectives, the following research questions have been formulated:

1. *What impact do IMQ*,* SRQ*,* and STQ have on the CCA by SMEs?*
2. *How does RLS mediate the relationship between CCA and IMQ*,* SRQ*,* and STQ?*
3. *In what ways does the relationship between quality considerations and the CCA get tempered by EDC?*
4. *What is the normalised importance of the factors influencing SME managers’ decisions to adopt cloud computing?*

This study is significant for both industry and academia as it provides a comprehensive understanding of the factors influencing CCA in SMEs. It highlights the mediating role of RLS, which underscores the necessity of a well-organised legal and policy framework. The moderating role of EDC offers valuable insights into how management expertise impacts decisions regarding technology adoption. Furthermore, it presents evidence-based recommendations for policymakers to create regulations that encourage cloud adoption. It also offers actionable strategies for SME managers to enhance their digital capabilities and leverage cloud computing to improve their competitiveness and growth. By addressing methodological gaps through the integration of predictive analytics and focusing on underexplored moderating and mediating mechanisms, this study contributes novel theoretical insights and practical guidance for SME digital transformation in emerging economies.

The article is organised as follows for the rest of it. The theoretical framework that underpins the formulation of hypotheses is described in the next section, which also provides a summary of previous research. Next, the selected methodology is presented. The results of the experiment are shown and explained in the following section. The study results are then examined. The study’s limitations and potential avenues for further investigation are finally mentioned.

## Theoretical framework and hypotheses development

### Theoretical framework

#### Technology-organization-environment

The TOE framework, which provides a comprehensive viewpoint on cloud computing adoption by SMEs, serves as the foundation for this study. The TOE framework, which was put forth by Tornatzky^8^, describes how organisational, technical, and environmental elements interact to affect how organisations adopt and use new technologies^9^. This study looks at IMQ, SRQ, and STQ as important factors that influence the CCA in a technological setting. STQ dictates the operation and performance of cloud platforms^10^, SRT service quality shows the effectiveness and responsiveness of cloud providers^11,12^, and high-quality information guarantees data accuracy and reliability^13^. Because they address performance, security, and usability issues, these technological elements directly affect SMEs’ perceptions of and desire to use cloud computing.

EDC is emphasised in the organisational environment as a moderating factor in the adoption process. SME managers’ evaluation, deployment, and optimisation of cloud technologies within their companies are greatly influenced by their level of digital competency^14^. Managers with higher levels of digital competency are better able to minimise perceived risks, make well-informed decisions, and optimise the advantages of cloud adoption^15^. The environmental context emphasises the mediating function of RLS, acknowledging that government policies, data protection regulations, and legal frameworks affect the security and trustworthiness of cloud adoption^16^. A robust regulatory framework can reduce apprehensions and encourage SMEs to adopt cloud computing. By combining these aspects, the TOE framework offers a methodical way to examine how SMEs use cloud computing, providing important insights into how technology, management skills, and regulatory frameworks interact.

### Hypotheses development

#### Information quality and cloud computing adoption

IMQ has a direct impact on decision-making, usability, and trust; it is crucial in determining how SMEs adopt cloud computing^17^. For companies that depend on cloud-based systems for data processing, storage, and real-time decision-making, high-quality information guarantees correctness, completeness, relevance, and consistency^18^. Cloud adoption may be discouraged by poor information quality, such as erroneous or out-of-date data, which can result in operational inefficiencies and elevated risks^19^. On the other hand, SMEs are more likely to view cloud computing as a useful and secure technology when cloud service providers deliver high levels of data integrity and reliability^4^. Additionally, cloud systems with strong information management features boost organisational effectiveness, enabling companies to streamline processes and enhance client interactions^1,20^. Recent studies further emphasise that as digital ecosystems evolve, information quality plays an increasingly strategic role in shaping SMEs’ digital transformation readiness, particularly when firms rely on cloud-enabled analytics and AI-driven decision systems^21–23^. These works highlight that high-quality data not only supports operational tasks but also enhances predictive capabilities and innovation potential within SMEs. Given how crucial accurate and reliable information is to the adoption of cloud computing, the following hypothesis is formulated:

##### H1a:

*IMQ has a positive impact on CCA in SMEs.*

##### H1b:

*IMQ has a positive impact on RLS.*

#### Service quality and cloud computing adoption

SRQ is a key factor in determining the adoption of cloud computing, which affects SMEs’ opinions of cloud service providers’ reliability, responsiveness, and general level of satisfaction^24^. Effective, scalable, and secure cloud solutions are guaranteed by high service quality, allowing companies to continue operating uninterrupted^25^. Data security, service availability, technical assistance, and provider response are important components of cloud computing service quality^26^. SMEs are more likely to use cloud technology when they receive fast support and consistent service performance^27^. Conversely, inadequate service quality, including frequent outages, a lack of customer care, or security flaws, breeds doubt and deters companies from moving to cloud-based systems^28^. Recent studies also show that service quality increasingly influences SMEs’ digital confidence, especially as cloud platforms integrate AI-driven service automation and predictive maintenance tools, which enhance user experience and reduce perceived operational risks^29,30^. Moreover, cloud ecosystems that offer proactive service analytics and continuous monitoring are found to reinforce SMEs’ trust and long-term commitment to cloud adoption. In light of this, the following hypothesis is put forth:

##### H2a:

*SRQ has a positive impact on CCA in SMEs.*

##### H2b:

*SRQ has a positive impact on RLS.*

#### System quality and cloud computing adoption

STQ affects the effectiveness, security, and general performance of cloud-based systems; it is a significant determinant of SMEs’ adoption of cloud computing^31^. According to Kommisetty and Abhireddy^32^, simplicity of use, system dependability, adaptability, and smooth integration with current business procedures are characteristics of a high-quality cloud system. SME adoption of cloud computing as a strategic tool is more likely when they believe that cloud systems are reliable, easy to use, and able to manage complicated business operations^2^. Büyüközkan, Uztürk^33^ assert that adoption decisions are significantly influenced by the system’s speed, uptime, and security characteristics. Cloud adoption becomes more appealing when SMEs have faith in a well-designed cloud system that reduces downtime, protects data privacy, and offers scalable solutions^34^. Adoption is hampered, though, if system quality is seen as insufficient and is typified by sluggish processing rates, frequent technological malfunctions, or inadequate data security^35^. Recent research also emphasises that advanced cloud architectures integrating edge computing, AI-driven optimisation, and automated security controls significantly enhance perceived system quality, thereby improving SMEs’ confidence in adopting cloud platforms^36–38^. Considering how crucial system quality is to cloud computing, the following hypothesis is formulated:

##### H3a:

*In SMEs*,* STQ positively affects CCA.*

##### H3b:

*STQ has a positive impact on RLS.*

#### Regulatory support as a mediator

RLS significantly mediates the adoption of cloud computing by SMEs^39^, providing a structured legal and policy framework that enhances compliance, security, and confidence. Governments and regulatory agencies influence cloud adoption by implementing cybersecurity rules, data protection laws, and compliance guidelines that reduce the perceived risks of cloud-based solutions^40,41^. Cloud service providers uphold high standards of data protection, service reliability, and transparency thanks to robust legislative frameworks, which in turn encourage SMEs to adopt cloud technology^42^. Conversely, uncertainty arising from unclear regulations or lax enforcement may deter companies from making a full switch to cloud-based systems due to concerns about operational risks, legal ramifications, and data breaches^43^. Furthermore, by lowering financial and legal barriers, regulatory incentives like tax breaks, subsidies, and compliance assistance can promote cloud usage among SMEs even more^44^. Recent empirical research also highlights that in developing economies, regulatory support increasingly interacts with digital governance practices, such as national cloud policies, cross-border data flow regulations, and sector-specific compliance mandates, to shape SMEs’ confidence in cloud ecosystems^45,46^. The following hypothesis is articulated in light of the crucial role that RLS plays in risk mitigation and CCA:

##### H4a:

*RLS has a positive impact on CCA.*

##### H4b:

*RLS mediates the relationship between IMQ and CCA.*

##### H4c:

*RLS mediates the relationship between SRQ and CCA.*

##### H4d:

*RLS mediates the relationship between STQ and CCA.*

#### Entrepreneurial digital competence as a moderator

**Fig. 1:**
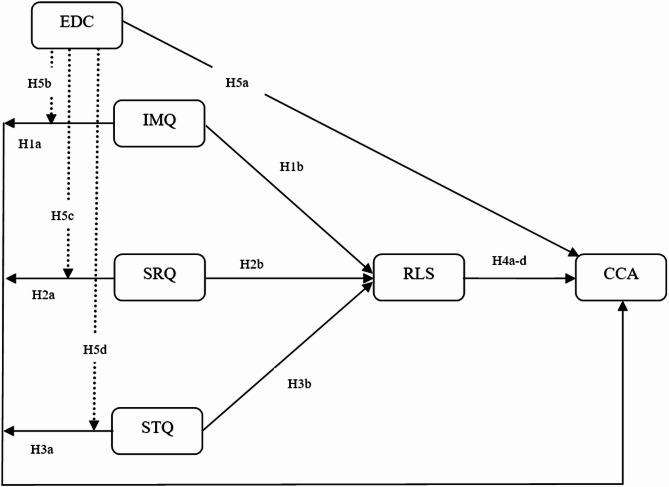
Theoretical framework.

EDC is a key moderator of CCA by SMEs since it measures business managers’ comprehension, assessment, and integration of digital technology^47^. AlDaajeh, Saleous^48^ state that digital competence includes abilities, including cybersecurity awareness, technological literacy, and the capacity to use digital technologies for innovation and company expansion. According to Saratchandra and Shrestha^49^, SMEs with managers who possess a high level of digital competency are more likely to understand the advantages of cloud computing, effectively oversee cloud-based operations, and reduce related risks. Digital competency also helps managers make better decisions by allowing them to evaluate cloud service providers’ reliability, comprehend regulatory compliance standards, and maximize cloud solutions for business scalability^50^. Conversely, managers who lack digital competency may find it difficult to handle operational complexity, data security issues, and technology implementation^5^. Therefore, SME managers’ level of digital competency determines the extent to which information, service, and system quality affect the adoption of cloud computing. In light of this, the following hypotheses are put forth: Finally, Fig. 1 shows the theoretical framework.

##### H5a:

*EDC has a positive impact on CCA.*

##### H5b:

*EDC moderates the relationship between IMQ and CCA in SMEs*.

##### H5c:

*EDC moderates the relationship between SRQ and CCA in SMEs*.

##### H5d:

*EDC moderates the relationship between STQ and CCA in SMEs*.

## Research methodology

### Population and sampling technique

This study investigates the relationship between CCA in Pakistani SMEs and IMQ, SRQ, and STQ, with EDC as a moderator and RLS as a mediator. Following a positivist research philosophy, a structured survey questionnaire was employed as part of a quantitative research design. The target population comprised SME managers directly involved in technology adoption decisions, as their expertise and roles ensure informed, relevant responses. SMEs were defined according to the Small and Medium Enterprises Development Authority (SMEDA), where small firms employ up to 50 people and medium firms employ 51–250 employees^51,52^. A purposive sampling technique was used to identify respondents with adequate knowledge of cloud implementation processes^53^. This approach helped ensure that the collected data accurately reflects the viewpoints of key decision-makers^54,55^. To enhance representativeness, questionnaires were distributed proportionally across major industrial sectors (e.g., textile, chemical, manufacturing, IT, retail, and services) and across key geographic regions (Punjab, Sindh, Khyber Pakhtunkhwa, and Balochistan).

### Data collection and measurement instruments

After completing the questionnaire, we consulted two experts to assess its structure, relevance, and clarity. Changes were made to improve the validity of the questionnaire based on their suggestions. A combination of online questionnaires (sent by email and WhatsApp) and in-person meetings with SME managers was used to collect data to increase the response rate^56^. Informed consent was obtained for data collection. This study was approved by the relevant institutional ethics committee. All procedures involving human participants were conducted in accordance with the ethical standards of the institutional and/or national research committee and the 1964 Helsinki Declaration and its later amendments, or comparable ethical standards.

A pilot study involving 30 participants was conducted before the full-scale survey to evaluate the instrument’s efficacy and reliability. During the primary data collection phase (November-December 2024), a total of 700 questionnaires were distributed to SME managers responsible for technology adoption decisions. The questionnaires were proportionally allocated across major industrial sectors in Pakistan (textile, chemical, manufacturing, IT, retail, and services) and key geographical regions, including Punjab, Sindh, Khyber Pakhtunkhwa, and Balochistan, to enhance representativeness. In total, 423 responses were received, yielding a 60.4% response rate. After a detailed data cleaning process, 367 valid responses were retained for analysis. The final sample size exceeded the minimum requirement for PLS-SEM, following the 10-times rule, thereby ensuring sufficient statistical power and model stability^57^. Specifically, responses were removed if they had more than 20% missing data, exhibited straight-lining patterns across multiple items (i.e., the same response repeated consecutively for five or more items), or contained inconsistent responses where contradictory answers were provided for logically linked questions. This systematic approach ensured that only high-quality, reliable data were used for analysis, while minimising potential bias against less digitally sophisticated respondents. While textile (25.07%) and chemical firms (21.25%) were more represented, this reflects the structural composition of Pakistan’s SME landscape. To assess non-response bias, an early–late respondent comparison (Armstrong & Overton, 1977) was conducted, showing no significant differences (*p* > 0.05). All procedures adhered to relevant research guidelines and ethical standards.

The questionnaire was split into two sections: the first part collected participants’ demographic data, while the second covered the study’s components. The established scales used to measure the variables in this study were appropriately adjusted to meet the study’s goals and context. Each component was assessed using a five-point Likert scale, which went from 1, denoting “Strongly Disagree,” and 5, “Strongly Agree,” in addition to demographic variables^58,59^. A four-times scale of CCA was adapted from Asif and Sarwar^60^. RLS was measured by using four items taken from He, Walker^61^. Furthermore, four items were used to assess IMQ, adapted from Kuo and Lee^62^, while three items were used to assess SRQ, adapted from Lee and Wu^63^, and three items for STQ, adapted from Gorla, Somers^64^.

### Data analysis

A two-stage method was used to validate hypotheses and develop a prediction model for the following reasons: Smart-PLS offers significant advantages for handling complex models, non-normally distributed data, and small sample sizes^65^. Additionally, data normality testing is necessary for AMOS as opposed to Smart-PLS^66^. Given the foregoing, this study uses Smart-PLS-4 (4.1.0.9) for data analysis, thereby eliminating the need for data normality testing. SEM is a theory-driven method that can only identify linear correlations between exogenous and endogenous variables by using a compensating model, where an increase in one variable could offset a drop in another^67^. Compared to SEM, ANN can use non-compensatory models to capture both linear and non-linear relationships^68^, leading to higher prediction accuracy. Furthermore, ANN is better suited for prediction than hypothesis testing because of its “black box” character^69^. Finally, the results from SEM can be further validated using ANN analysis. As a result, this work integrates a hybrid approach to hypothesis testing and prediction, combining the advantages of both.

Specifically, in the first phase, SEM was used to reveal the impacts of IMQ, SRQ, STQ, RLS, and EDC on CCA. In the second phase, the crucial variables were utilized as ANN input neurons to predict the CCA in Pakistani SMEs using IBM SPSS. This two-phase method offers a thorough analysis that capitalizes on the advantages of both SEM and ANN approaches, enabling a thorough investigation of the constructs and their predictive capacity.

## Analysis and results

### Descriptive analysis and common method bias (CMB)

The descriptive analysis of the sample, shown in Table 1, indicates that SMEs are represented across a variety of industrial sectors, firm age groups, employee sizes, and certification statuses. The greatest share is made up of the textile industry (25.07%), which is followed by the chemical (21.25%), pharmaceutical (13.62%), and steel (10.63%) sectors. Other industries that make substantial contributions include IT & software (10.90%), food & beverage (10.08%), and electronics (8.45%). A well-established position in the market is evident from the fact that the majority of enterprises have been in operation for more than 20 years, with 32.43% having been in operation for more than 30 years and 30.79% for 20–30 years. The majority of businesses (32.15%) have 150–200 employees, followed by 28.88% with 200–250 employees. This indicates that medium-sized businesses are significantly represented in the workforce. Finally, 40.87% of businesses are not certified, suggesting potential areas for adopting quality standards, whereas 59.13% are ISO certified, indicating a significant predisposition towards quality management standards. This sample distribution ensures a well-balanced dataset for examining CCA among Pakistani SMEs.

The single source of data collection poses a major risk to data quality. To lower the chance of CMB, procedures and statistical safeguards were implemented^70^. The intended goal of this study was stated on the questionnaire for procedural remedies. As a statistical correction, Harmon’s single-factor test was applied to every research variable. The results showed that the maximum variance (46%) that could be ascribed to a single component was less than the recommended threshold value of 50%^70^. Kock^71^ and Kock and Lynn^72^ predicted that all items’ variance inflation factor (VIF) values in the PLS-SEM experiments would be ≤ 3.3, which would mean that the framework is free of CMB. Every study item in the current investigation met the minimal VIF threshold values (see Table 2).

**Table 1:** Descriptive analysis.

Category	Frequency	%
Industrial sector		
Textile	92	25.07
Chemical	78	21.25
Pharmaceutical	50	13.62
Steel	39	10.63
IT & Software	40	10.90
Food & Beverage	37	10.08
Electronics	31	8.45
**Firm age (Years)**		
Less than 10	49	13.35
10–20	86	23.43
20–30	113	30.79
Above 30	119	32.43
**Number of employees**		
Less than 100	51	13.90
100–150	92	25.07
150–200	118	32.15
200–250	106	28.88
**ISO certification**		
ISO certified	217	59.13
Non-ISO certified	150	40.87

### Assessment of measurement model

#### Reliability and convergent validity

As recommended by Hair, Sarstedt^73^, several tests, including Cronbach’s alpha (α) and composite reliability (CR), were conducted to guarantee the reliability of the data. However, according to Hair, Sarstedt^57^, CR is a more trustworthy metric than Cronbach’s α. Although a CR value of 0.60 to 0.70 is also regarded as acceptable, a value of ≥ 0.70 is deemed sufficient^57^. According to Table 2, all research constructs have CR over 0.70, indicating that they are all suitable for additional analysis. Measures such as average variance extracted (AVE) and outer indicator loading were used based on previous research to assess the convergent validity^57^. A threshold value of ≥ 0.708 is regarded as outstanding in terms of indicator loading, while a value in the range of 0.4 and 0.7 is regarded as acceptable^57^. According to Hair, Sarstedt^57^, a threshold value of 0.5 has been proposed as appropriate for AVE to confirm convergent validity.

**Table 2:** Reliability and convergent validity.

Dimensions	Items	Loadings	VIF	α	CR	AVE
Cloud computing adoption	CCA1	0.830	2.067	0.861	0.906	0.707
	CCA2	0.853	2.185			
	CCA3	0.811	1.858			
	CCA4	0.868	2.243			
Regulatory support	RLS1	0.854	2.206	0.859	0.904	0.703
	RLS2	0.843	2.154			
	RLS3	0.838	1.987			
	RLS4	0.818	1.810			
Information quality	IMQ1	0.831	1.971	0.875	0.915	0.728
	IMQ2	0.817	1.928			
	IMQ3	0.885	2.682			
	IMQ4	0.878	2.601			
Service quality	SRQ1	0.880	2.075	0.858	0.913	0.778
	SRQ2	0.870	2.122			
	SRQ3	0.896	2.282			
System quality	STQ1	0.859	1.704	0.803	0.883	0.717
	STQ2	0.841	1.715			
	STQ3	0.839	1.764			
Entrepreneurial digital competence	EDC1	0.768	1.417	0.745	0.854	0.661
	EDC2	0.829	1.603			
	EDC3	0.840	1.484			

#### Discriminant validity

The discriminant validity is assessed using a variety of metrics based on PLS-SEM investigations. The Heterotrait-Monotrait (HTMT) ratio of correlation, according to Hair, Sarstedt^57^, is a more suitable method than others (such as cross-loading and Fornell-Larker). Henseler, Ringle^74^ state that other metrics cannot prove discriminant validity in PLS-SEM. Thus, the HTMT criterion was applied. Discriminant validity is demonstrated by a threshold HTMT value of 0.90 for comparable constructs and less than 0.85 for different constructs^57^. The HTMT results show that each construct’s HTMT value is less than 0.85 in Table 3.

**Table 3:** Discriminant validity.

Constructs	1	2	3	4	5	6
1. CCA						
2. EDC	0.577					
3. IMQ	0.795	0.608				
4. RLS	0.849	0.685	0.846			
5. SRQ	0.803	0.659	0.832	0.842		
6. STQ	0.561	0.679	0.606	0.583	0.598	

### Structural model and hypothesis analysis

#### Direct analysis

Henseler, Ringle^74^ proposed the bootstrapping technique with 5000 resamples to assess the structural model and hypothesis analysis. Additionally, Hahn and Ang^75^ argued that researchers shouldn’t base their acceptance or rejection of a hypothesis only on the p-value. They recommended that when analyzing hypotheses, the confidence interval (CI) be presented. As a result, CI determined whether a hypothesis was accepted or rejected.

Table 4 illustrates the findings of the hypothesis test and offers important new information about how the variables under investigation relate to one another. Supporting H1a and H1b, the results show that IMQ significantly and favorably affects CCA (β = 0.215, *p* = 0.000) and RLS (β = 0.423, *p* = 0.000). H2a and H2b are accepted as a result of SRQ’s beneficial effects on CCA (β = 0.174, *p* = 0.008) and RLS (β = 0.404, *p* = 0.000). H3a is rejected because STQ has no significant effect on CCA (β = 0.071, *p* = 0.119), but it considerably improves RLS (β = 0.072, *p* = 0.047), which supports H3b. Furthermore, it is discovered that RLS considerably improves CCA (β = 0.397, *p* = 0.000), supporting H4a. On the other hand, H5a is rejected since EDC does not significantly affect CCA (β = 0.006, *p* = 0.886). These findings underline the crucial role that IMQ and SRQ play in the adoption of cloud computing. To comprehend their limited impact on the adoption process, STQ and EDC need more research.

**Table 4:** Hypothesis testing.

Relation	β-value	t-value	CI [5.0% − 95.0%]	*p*-value	Decision
H1a: IMQ → CCA	0.215	3.545	[0.084–0.321]	0.000	Accepted
H1b: IMQ → RLS	0.423	6.330	[0.293–0.554]	0.000	Accepted
H2a: SRQ → CCA	0.174	2.654	[0.050–0.307]	0.008	Accepted
H2b: SRQ → RLS	0.404	6.021	[0.271–0.531]	0.000	Accepted
H3a: STQ → CCA	0.071	1.558	[−0.022–0.155]	0.119	Rejected
H3b: STQ → RLS	0.072	1.991	[0.001–0.142]	0.047	Accepted
H4a: RLS → CCA	0.397	6.154	[0.274–0.524]	0.000	Accepted
H5a: EDC → CCA	0.006	0.144	[−0.075–0.093]	0.886	Rejected

#### Mediation analysis

Table 5 presents the results of the mediation study showing that the association between CCA, IMQ, SRQ, and STQ is mediated by RLS. H4b and H4c are accepted since the results show that RLS significantly mediates the link between SRQ and CCA (β = 0.160, t = 4.434, *p* = 0.000) and between IMQ and CCA (β = 0.168, t = 4.145, *p* = 0.000). These findings imply that improved IMQ and SRQ strengthen regulatory backing, which helps SMEs adopt cloud computing. H4d is rejected because the RLS mediation effect between STQ and CCA (β = 0.029, t = 1.866, *p* = 0.062) is not statistically significant. This suggests that although RLS may be influenced by STQ, the adoption of cloud computing is not much impacted indirectly. All things considered, these results highlight how crucial regulatory backing is to enhancing the influence of IMQ and SRQ on the CCA, whereas STQ plays a relatively small part in this regard.

**Table 5:** Mediation analysis.

Relation	β-value	t-value	*p*-value	Decision
H4b: IMQ → RLS → CCA	0.168	4.145	0.000	Accepted
H4c: SRQ → RLS → CCA	0.160	4.434	0.000	Accepted
H4d: STQ → RLS → CCA	0.029	1.866	0.062	Rejected

#### Moderation analysis

Table 6 presents the moderation analysis examining the links between CCA, IMQ, SRQ, and STQ in EDC. EDC significantly moderates the association between SRQ and CCA (β = 0.141, t = 2.875, *p* = 0.004) and between IMQ and CCA (β = 0.158, t = 2.740, *p* = 0.006), which supports H5b and H5c. These results imply that entrepreneurs with greater digital competency are better able to leverage high-quality information and services to successfully implement cloud computing. H5d is rejected because the moderating impact of EDC between STQ and CCA (β = −0.031, t = 0.814, *p* = 0.416) is not significant. This suggests that the association between STQ and CCA is not influenced by EDC, suggesting that other factors may be more important in this interaction. While STQ is less reliant on entrepreneurs’ digital competency, these findings generally emphasise the significance of digital competency in augmenting the influence of information and service quality on cloud adoption.

**Table 6:** Moderation analysis.

Relation	β-value	t-value	*p*-value	Decision
H5b: EDC * IMQ → CCA	0.158	2.740	0.006	Accepted
H5c: EDC * SRQ → CCA	0.141	2.875	0.004	Accepted
H5d: EDC * STQ → CCA	−0.031	0.814	0.416	Rejected

### Artificial neural network analysis

This section summarises the findings and explains why ANN is used. Nevertheless, the linear relationship between independent and dependent variables is captured using SEM. However, its applicability is insufficient to fully explain the complex dynamics of decision-making processes^76,77^. Additionally, SEM is based on the compensating assumption, which suggests that a decrease in one of the exogenous components of the model can be compensated for by an increase in another^76^. A hybrid SEM-ANN approach is employed to overcome this challenge and capture linear and nonlinear correlations inside a non-compensatory architecture^77^. The hybrid SEM-ANN methodology represents a significant advancement in research methods.

This study utilises the ANN technique to assess the relationship between each predictor variable and the dependent variable, owing to its advantages and widespread acceptance. The ANN algorithm consists of three layers: input, hidden, and output^78^. A multi-layer perceptron using feedforward and backpropagation was utilised in the ANN analysis. Additionally, a tenfold cross-validation method was implemented to reduce the possibility of overfitting^76^. Previous studies indicated that training and testing were performed using a 90:10 data split^78,79^. The “root mean square error” (RMSE) values were calculated (Table 7), demonstrating that the analysis resulted in a well-fitting model^79^.

**Table 7:** RMSE values.

Neural networks	Training			Testing			Total sample
	*N*	SSE	RMSE	*N*	SSE	RMSE	
1	330	5.959	0.134	37	0.833	0.150	367
2	330	6.196	0.137	37	0.627	0.130	367
3	326	6.014	0.136	41	0.627	0.124	367
4	321	5.952	0.136	46	1.158	0.159	367
5	333	6.347	0.138	34	0.401	0.109	367
6	325	6.091	0.137	42	0.651	0.124	367
7	331	5.841	0.133	36	0.294	0.090	367
8	323	5.633	0.132	44	0.624	0.119	367
9	330	5.517	0.129	37	0.666	0.134	367
10	330	5.756	0.132	37	0.266	0.085	367
Mean		5.931	0.134	Mean	0.615	0.122	
S. D		0.340	0.003	S. D	0.249	0.022	

As indicated in Table 8, sensitivity analysis was performed to evaluate each input neuron’s predictive ability. An ANN model is employed for sensitivity analysis, which evaluates the relative importance of the independent variables: RLS, IMQ, SRQ, STQ, and EDC in predicting CCA. The findings reveal that RLS has the highest normalised value at 100%, indicating that SME managers’ decisions to adopt cloud computing are most strongly influenced by this factor. This suggests that the likelihood of adopting cloud computing increases significantly when a strong legal framework is in place. IMQ is considered the second most important factor influencing cloud adoption, with a significance rating of 63.22%, following closely behind RLS. SRQ also plays a significant role in adoption decisions, with a rating of 56.17%, indicating that effective cloud services positively impact adoption choices. STQ has a relevance rating of 35.19%, suggesting that while technical performance is important, other factors are more crucial when making decisions. Finally, EDC has the lowest normalised relevance at 27.17%, indicating that although digital skills are beneficial, they do not primarily drive cloud adoption.

**Table 8:** Sensitivity analysis.

Neural network	EDC	IMQ	RLS	SRQ	STQ
1	0.144	0.211	0.325	0.194	0.126
2	0.084	0.239	0.337	0.189	0.151
3	0.107	0.227	0.320	0.230	0.117
4	0.249	0.238	0.281	0.217	0.016
5	0.118	0.195	0.269	0.211	0.206
6	0.043	0.263	0.394	0.182	0.118
7	0.025	0.236	0.423	0.175	0.142
8	0.021	0.194	0.521	0.175	0.088
9	0.029	0.220	0.449	0.170	0.132
10	0.034	0.221	0.374	0.222	0.149
Average importance	0.085	0.224	0.369	0.197	0.125
Normalized importance (%)	27.17	63.22	100.0	56.17	35.19

## Discussion and conclusion

This study aims to investigate the factors that influence CCA among SMEs in Pakistan. It particularly focuses on the roles of IMQ, SRQ, and STQ. Additionally, the study considers RLS as a mediating variable and explores how EDC may enhance these relationships. To achieve these objectives, a hybrid approach combining SEM and ANN was employed. SEM was used to examine the proposed relationships, while ANN assessed non-linear relationships and determined the relative importance of each predictor in cloud adoption.

The results indicate that the CCA is heavily influenced by IMQ and SRQ, highlighting their importance in facilitating cloud adoption among SMEs. These findings align with the research of Putri^18^ and Alkhasawneh and Khasawneh^19^, who noted that SMEs’ decision to adopt cloud computing is primarily driven by the need for reliable, secure, and high-quality cloud services. This notion is further reinforced by the TOE framework proposed by Tornatzky^8^, which asserts that the decision to adopt new technologies is directly influenced by the technological characteristics of the services, including information accuracy and service reliability.

STQ was found to have no significant direct influence on CCA, a result that diverges from much of the prior literature. This unexpected outcome becomes meaningful when viewed in the context of Pakistani SMEs. Unlike firms in more technologically mature environments, many SMEs in Pakistan rely extensively on external IT vendors, cloud intermediaries, or informal IT consultants who manage system configuration, integration, and maintenance on their behalf. As noted by Jiao, Wu^80^, outsourcing technical functions can substantially reduce the salience of system-related attributes in adoption decisions. In such settings, decision-makers are less exposed to system complexities and therefore evaluate cloud services primarily based on information reliability, service responsiveness, trust, and regulatory compliance, rather than system design or integration features.

Moreover, the SME ecosystem in Pakistan is characterised by limited in-house digital expertise, leading entrepreneurs to prioritise service quality and institutional assurances over system functionality. This dynamic supports the argument of Büyüközkan, Uztürk^33^, who contend that in environments with low technical capacity, perceived risks, service reliability, and compliance pressures may overshadow system-related considerations. Thus, the insignificance of system quality in this study may reflect a structural characteristic of the Pakistani SME context, in which system complexity is effectively offloaded to external actors, thereby making system quality a less influential determinant of cloud computing adoption.

RLS plays a significant role in mediating the relationships among IMQ, SRQ, and CCA, as indicated by the mediation study. This suggests that laws related to data protection, compliance, and government regulations are crucial for encouraging CCA among SMEs. Mishra, Alzoubi^40^ and Wang, Asif^41^ noted that strong legal frameworks and regulatory support enhance trust in cloud technology, which, in turn, leads to higher adoption rates among SMEs. The mediation effect of RLS on the relationship between STQ and CCA was found to be statistically insignificant. This implies that regulatory actions alone may not be sufficient to resolve system quality issues, such as usability or integration problems.

Managers of SMEs with higher digital competency are better positioned to leverage information quality and service reliability when adopting cloud solutions. The moderation analysis shows that EDC enhances the impact of IMQ and SRQ on CCA. Yahya, Shukla^50^ assert that EDC is a key facilitator of digital transformation, which enables organisations to effectively incorporate cloud solutions into their operations. However, the lack of significance in the relationship between EDC and STQ indicates that system quality’s impact on cloud adoption is not solely amplified by EDC. This finding does not align with Saratchandra and Shrestha^49^, who noted that IT professionals often manage or outsource technical system quality, which reduces the direct influence of management’s digital competency on the barriers to system adoption.

The significance of various research factors in predicting CCA is illustrated by an ANN-based sensitivity analysis. Analysis identifies regulatory support as the most significant predictor of cloud computing adoption among Pakistani SMEs, with a normalized importance of 100%. A key aspect driving this effect is compliance with data protection laws, which provides firms with legal assurance regarding the storage, processing, and security of sensitive business and customer information. In the Pakistani SME context, clear and enforceable data protection regulations reduce perceived risks associated with cloud adoption and enhance trust in cloud service providers. Practically, this finding highlights the importance of strengthening data protection frameworks, ensuring compliance guidance is accessible to SMEs, and promoting awareness of legal responsibilities. By focusing on data protection as a central regulatory mechanism, policymakers can effectively facilitate cloud adoption and foster a safer, more reliable digital ecosystem for SMEs. IMQ accounts for 63.22%, while SRQ accounts for 56.17%; both are important considerations. Additionally, findings indicate that while management digital skills and technical system quality are important, they are not the primary drivers for cloud adoption in SMEs.

### Theoretical implications

This study extends the TOE framework by demonstrating that traditional quality factors do not operate uniformly within SME cloud adoption decisions. While information quality and service quality significantly influence adoption, system quality shows no direct effect, suggesting that SMEs increasingly prioritise externally provided digital capabilities, trust, and regulatory assurances over technical system features. This contextual insight contributes to the DeLone and McLean IS Success Model by showing that, in environments with limited internal digital expertise, system attributes may be overshadowed by external support mechanisms.

Furthermore, the significant mediating role of regulatory support highlights the growing influence of environmental and institutional pressures on digital adoption, challenging the assumptions of TAM and UTAUT that emphasise individual perceptions of usefulness and ease of use. Instead, this study demonstrates that SME adoption decisions are shaped by broader digital ecosystems, regulatory structures, and relational networks, aligning with emerging perspectives on trust-based and B2B technology adoption.

Importantly, this study advances theoretical discussions on digital transformation by acknowledging the evolving role of generative AI within cloud platforms. Generative AI capabilities, such as automated analytics, intelligent automation, and real-time decision support, are increasingly embedded in modern cloud services and may act as future moderators or mediators of adoption behaviour. Incorporating generative AI into cloud adoption models can enhance the explanatory power of TOE-based frameworks by recognising that SME decisions are no longer influenced solely by traditional quality dimensions but by AI-enabled value creation, resilience, and sustainability potential. This aligns with recent research (e.g., studies on generative AI for sustainable supply chains) and opens pathways for integrating emerging AI-driven capabilities into technology adoption theory.

### Practical implications

The findings of this study offer several actionable insights for SME managers, cloud service providers, and policymakers seeking to enhance cloud computing adoption and advance digital transformation. For SME managers, prioritising relational support and system reliability is crucial. Managers can achieve this by partnering with cloud service providers to access training programs, establishing internal cloud champions to lead digital initiatives, and leveraging peer networks or industry associations for knowledge sharing. These strategies help SMEs navigate system complexity, reduce perceived risks, and build confidence in cloud technologies.

Cloud service providers can support adoption by offering tailored consultancy, stepwise implementation plans, and customer training, enabling SMEs to gradually adopt cloud solutions and manage costs effectively. Scalable solutions that allow incremental adoption can reduce upfront expenses while facilitating smoother digital transformation. For policymakers, the study highlights the importance of regulatory support. Specific policy actions include providing tax incentives for cloud adoption, ensuring data localisation flexibility for SMEs, and funding digital literacy programs or subsidised training initiatives. By creating supportive regulatory and institutional environments, policymakers can mitigate adoption barriers and encourage wider SME participation in cloud-enabled digital ecosystems. Collectively, these strategies emphasise a multi-level approach, combining managerial initiatives, provider support, and targeted policy measures, to foster successful and sustainable cloud adoption among SMEs.

### Limitations and future research directions

Notwithstanding its contributions, this study has several limitations. First, the cross-sectional design restricts the ability to draw causal inferences from the observed relationships. The associations identified between information quality, service quality, and cloud computing adoption should not be interpreted as definitive causal pathways. Firms already engaged in cloud use may develop stronger expectations and greater awareness of information and service quality over time, potentially leading to reverse causality. Recent research adopting a systems perspective demonstrates that digital adoption, digital drive, and digital culture often interact in reinforcing feedback loops, underscoring the need for longitudinal research to capture these dynamic, reciprocal relationships. Future studies should therefore employ longitudinal or panel designs to observe how cloud adoption behaviours evolve and how digital competencies and quality perceptions shift over time.

Second, the study focuses exclusively on SMEs in a single national context, which may limit the generalisability of its findings to regions with different technological infrastructures, institutional frameworks, or levels of digital transformation maturity. Additionally, the sample concentration in the textile and chemical industries may introduce sectoral bias, and future studies should ensure broader industry representation to enhance generalizability. Comparative studies across countries or regions could provide deeper insights into how contextual differences shape cloud adoption patterns.

Third, this study relies primarily on quantitative methods, which may not fully uncover the nuanced motivations and challenges faced by SME managers. Future research could integrate qualitative approaches, such as case studies or interviews, to enrich the understanding of organisational decision-making processes. Moreover, the measurement of EDC relied on three items emphasising operational digital skills, which may not fully capture strategic or managerial digital capabilities. Future research should adopt more comprehensive multi-dimensional scales to explore nuanced relationships between EDC and cloud adoption. Fourth, the data were collected during November-December 2024, coinciding with the fiscal year-end for many SMEs. This timing may have influenced responses, as firms could have been more or less conservative in their technology adoption decisions due to budget constraints. Future studies should account for potential period effects when collecting data.

Finally, while this study examines key quality-related antecedents and entrepreneurial digital competency, other potentially important factors, such as cybersecurity concerns, cost-benefit evaluations, organisational culture, leadership orientation, and technological readiness, were not included. In particular, cost considerations, including perceived affordability, pricing models, and return on investment, were not explicitly examined, which may be especially relevant for resource-constrained SMEs. The lack of a significant effect of system quality may reflect SMEs’ prioritisation of cost-effective services over advanced technical features. Exploring these variables, as well as their potential interactions with digital skills, would help develop a more comprehensive understanding of the conditions that facilitate or hinder cloud adoption and would support SMEs in designing more effective digital transformation strategies.                                                                                                                                                                                                           

## Data Availability

The data supporting the findings of this study are available upon reasonable request. Interested parties may contact Zhi Li at [lizhi@nbt.edu.ch] for access.
